# Prehospital Airway Management Method as a Predictor of Early and Definitive Survival in Patients After OHCA

**DOI:** 10.3390/jcm15145481

**Published:** 2026-07-13

**Authors:** Łukasz Suchanek, Magdalena Augustyn, Michał Wójcik, Damian Krysiak, Piotr Babik, Michał Ćwiertnia, Arkadiusz Stasicki, Michał Szlagor, Mieczysław Dutka, Marek Kawecki, Wioletta Waksmańska, Tomasz Ilczak

**Affiliations:** 1 Department of Emergency Medicine, Faculty of Health Sciences, University of Bielsko-Biala, 43-309 Bielsko-Biała, Poland; maugustyn@ubb.edu.pl (M.A.); miwojcik@ubb.edu.pl (M.W.); dkrysiak@ubb.edu.pl (D.K.); pbabik@ubb.edu.pl (P.B.); mcwiertnia@ubb.edu.pl (M.Ć.); astasicki@ubb.edu.pl (A.S.); mszlagor@ubb.edu.pl (M.S.); tilczak@ubb.edu.pl (T.I.); 2 Department of Biochemistry and Molecular Biology, Faculty of Health Sciences, University of Bielsko-Biala, 43-309 Bielsko-Biała, Poland; mdutka@ubb.edu.pl; 3 Department of Nursing, Faculty of Health Sciences, University of Bielsko-Biala, 43-309 Bielsko-Biała, Poland; mkawecki@ubb.edu.pl; 4 Department of Public Health, Faculty of Health Sciences, University of Bielsko-Biala, 43-309 Bielsko-Biała, Poland; wwaksmanska@ubb.edu.pl

**Keywords:** out-of-hospital cardiac arrest, airway management, endotracheal intubation, supraglottic airway devices

## Abstract

**Background and Objective:** Out-of-hospital cardiac arrest is associated with high mortality, with prolonged cerebral hypoxia being one of the leading causes of death. The primary objective of this study was to evaluate the probability of survival during hospitalization in OHCA patients and to compare survival outcomes based on the prehospital airway management method—specifically, endotracheal intubation versus supraglottic airway devices. **Material and Methods:** A retrospective analysis was conducted using medical records of patients admitted to the Emergency Department of the Voivodeship Hospital in Bielsko-Biala between 1 January 2020 and 28 February 2024. The study included 61 OHCA patients who achieved the return of spontaneous circulation and had complete documentation regarding their airway management. Early and definitive survival were evaluated using a logistic regression model (adjusted for age and sex), and survival probabilities over time were estimated using the Kaplan–Meier method. **Results:** The analysis revealed no statistically significant difference in early survival between patients managed with an endotracheal tube and those managed with an SGA device (RR = 0.80; *p* = 0.598). Similarly, no significant difference was observed for definitive survival to hospital discharge (RR = 1.13; *p* = 0.836). Kaplan–Meier survival curves indicated a sharp decline in overall survival probability during the initial days of hospitalization, but the log-rank test (*p* = 0.600) confirmed the lack of significant differences in survival trajectories between the two airway management groups. Patient age was the only statistically significant factor influencing early survival, with the probability decreasing by an average of 3% for each advancing year. **Conclusions:** The study did not demonstrate any superiority of the endotracheal tube over supraglottic airway devices regarding the in-hospital survival of OHCA patients. The decision regarding prehospital airway management should be individualized, taking into account the operator’s experience and the critical need to minimize interruptions in chest compressions during advanced life support.

## 1. Introduction

According to the latest scientific reports, the average incidence of out-of-hospital cardiac arrest in Europe is 55 per 100,000 inhabitants, with approximately 70% of cases occurring in a home environment. Research indicates that the average survival rate to hospital discharge is approximately 7.5%, ranging from 3.1% to 35% across European countries [1]. Out-of-hospital cardiac arrest is associated with high mortality, with prolonged cerebral hypoxia being one of the leading causes of death. A crucial aspect of cardiopulmonary resuscitation is its quality, which directly impacts patient survival. According to study results, the most critical factors influencing resuscitation efficacy are chest compressions and early defibrillation. However, when considering survival in cardiac arrest episodes, reversible causes of arrest—which are often difficult to identify in the prehospital setting—cannot be overlooked [2,3]. One such reversible cause is hypoxia. To effectively counteract it, the patient’s airway must be appropriately secured. Depending on the rescuer’s skill level, emergency medicine offers various airway management techniques. The 2025 European Resuscitation Council guidelines recommend starting with basic airway management techniques and progressing stepwise, according to the rescuer’s competence, until effective ventilation is achieved [4,5]. Endotracheal intubation should only be performed by rescuers with a high success rate and with the use of continuous waveform capnography. The expert consensus defines a high success rate as exceeding 95% within two intubation attempts [3]. The recommended supraglottic airway device is the i-gel [6,7]. The continuous development of emergency medical systems worldwide leads to the search for new diagnostic and therapeutic solutions aimed at reducing mortality in patients following prehospital cardiac arrest.

### Objectives

The primary objective of this study was to evaluate the overall probability of survival during hospitalization in patients following out-of-hospital cardiac arrest, and thus to characterize the temporal dynamics of mortality risk within this highly vulnerable population. Secondary objectives included a comparative assessment of survival outcomes based on the airway management method—specifically, endotracheal intubation versus the use of supraglottic airway devices. This comparison aimed to determine whether the choice of airway management technique influences the survival trajectory throughout the course of hospitalization.

## 2. Materials and Methods

This study was retrospective in nature. It was conducted based on an analysis of medical records of patients who experienced out-of-hospital cardiac arrest and were subsequently transported to the Emergency Department of the Voivodeship Hospital in Bielsko-Biala. The study received approval from the hospital administration (No. N.MO/10/2023). A positive opinion was also obtained from the Research Ethics Committee (No. 2025/01/5/E/5) regarding the compliance of this scientific project with ethical principles.

### 2.1. Medical Records Analysis

Medical records from the period of 1 January 2020 to 28 February 2024, were analyzed. According to the collected data, 144,213 patients were admitted to the ED during this period, of whom 79 had experienced OHCA (identified by ICD-10 code: I46.0—Cardiac arrest with successful resuscitation).

The following inclusion criteria were applied for the study:

- Out-of-hospital cardiac arrest with ROSC based on ICD-10 diagnosis.
- Patients transported to the Emergency Department of the Voivodeship Hospital in Bielsko-Biała.
- Presence of documentation regarding the airway management method used—either endotracheal intubation or supraglottic airway devices (such as i-gel, LMA, LT).
- Complete medical documentation at every stage of the data analysis.

The exclusion criteria included:

- Direct transport of the patient to specialized hospital wards (e.g., hemodynamics unit, operating theater) bypassing the ED.
- Incomplete hospital treatment records.
- Lack of information regarding the prehospital airway management method.
- Patients who, after initial diagnostics in the ED, were transferred to other referral centers.

### 2.2. Methods

To ensure the appropriate standard of data analysis, data were collected in a computerized system by hospital statisticians and made available to the researchers on a dedicated hospital workstation. After identifying the records and hospitalization numbers, physical medical records were accessed for detailed analysis. The documentation review was conducted in strict accordance with the established inclusion and exclusion criteria. Data entry into a dedicated matrix was performed exclusively by the research team members. All identifiable patient information was blinded and assigned unique identification numbers to prevent subsequent identification. The data were entered into a customized matrix stored on a password-protected portable storage device.

In the study documentation, survival was categorized as early and definitive based on the duration of the patient’s hospitalization. In this study:

- Early survival was defined as the absence of death until the day following admission. This timeframe was pragmatically chosen as a proxy for successful initial stabilization in the ED and subsequent admission to the Intensive Care Unit, representing the critical initial phase of post-cardiac arrest syndrome management.
- Definitive survival referred to patients who were discharged from the hospital, regardless of their neurological status at the time of discharge.

The study analysis was conducted based on the established research hypotheses. Early and definitive survival were analyzed against explanatory variables, specifically the airway management method—endotracheal intubation versus supraglottic airway devices (i-gel, LMA, LT). Additionally, demographic characteristics such as age and gender were included in each analysis as confounding variables. All collected material was subjected to statistical analysis.

An artificial intelligence tool (specifically the Gemini 3.1 Pro model) was utilized in the preparation of this article for grammatical, stylistic, and punctuation corrections and bibliography formatting. The authors have reviewed and edited the output and take full responsibility for the content of this publication.

### 2.3. Statistical Analysis

Statistical analysis was performed using the R statistical programming language. The significance level was set at α = 0.05. The normality of continuous variable distributions was assessed using the Shapiro–Wilk test. Data are presented as means and standard deviations (SDs) or medians with the first and third quartiles (Q1–Q3), depending on whether the normality criteria were met.

To evaluate the relationship between the airway management method and patient survival, a logistic regression model with a probit link function was employed. This model allowed for the estimation of relative risk (RR) for the respective independent variables. To determine the effect size of the studied variables and their statistical significance, 95% confidence intervals (95% CIs) and *p*-values were calculated using the Wald test approximation. Patient survival served as the dependent variable, while the airway management method was the independent variable. Potential confounding variables, such as age and gender, were also included in the model.

The analyses were conducted using the R statistical language (version 4.3.1; R Core Team, 2023) on a Windows 10 Pro 64-bit system (build 19045), with the following packages: ggeffects (version 1.3.2; Lüdecke D, 2018), sjPlot (version 2.8.15; Lüdecke D, 2023), performance (version 0.10.8; Lüdecke D et al., 2021), report (version 0.5.7; Makowski D et al., 2023), ggstatsplot (version 0.12.1; Patil I, 2021), gtsummary (version 1.7.2; Sjoberg D et al., 2021), MASS (version 7.3.60; Venables WN, Ripley BD, 2002), readxl (version 1.4.3; Wickham H, Bryan J, 2023), dplyr (version 1.1.3; Wickham H et al., 2023), and psych (version 2.3.9; William Revelle, 2023).

The probability of survival was estimated using the non-parametric Kaplan–Meier method, which accounts for right-censored data and enables the construction of step-function survival curves. The time-to-event was defined as the period from hospital admission to death or censoring at the time of discharge or end of observation. Stratification by the type of airway device allowed for the generation of subgroup-specific survival curves. Differences between survival curves were assessed using the log-rank test, providing the chi-square statistic and the corresponding *p*-value under the null hypothesis of equivalent survival distributions. Confidence intervals (95% CIs) for survival estimators were determined using Greenwood’s formula as a variance approximation. Descriptive summaries, including the number at risk and the number of events at defined time points (days 1, 7, and 14), provided additional quantitative data. Due to the exploratory nature of the analysis, no corrections for multiple comparisons were applied, and a *p*-value < 0.05 was considered the threshold for statistical significance.

Additional analyses were performed using R (version 4.5.2; R Core Team, 2025) on Windows 11 Pro 64 bit (build 26100), using the packages report (version 0.6.2; Makowski D et al., 2023), ggsurvfit (version 1.2.0; Sjoberg D et al., 2025), survival (version 3.8.3; Therneau T, 2024), ggplot2 (version 4.0.1; Wickham H, 2016), and dplyr (version 1.1.4; Wickham H et al., 2023).

## 3. Results

After applying the inclusion and exclusion criteria, 61 patient records were qualified for the study. The detailed characteristics of the study group are presented in Table 1.

The baseline demographic characteristics and clinical outcomes of the study population (*n* = 61), categorized by the prehospital airway management strategy (ETI, *n* = 49; SGA, *n* = 12), are summarized in Table 2. The overall cohort had a median age of 66.0 years (Q1–Q3: 54.0–75.0) and was predominantly male (73.8%). There were no statistically significant differences between the ETI and SGA groups concerning baseline demographics, including age (*p* = 0.710) and sex distribution (*p* = 0.270). Furthermore, clinical outcomes such as early survival with subsequent ICU admission (49.0% in the ETI group vs. 58.3% in the SGA group; *p* = 0.749) and definitive survival to hospital discharge (10.2% vs. 8.3%; *p* > 0.99) did not differ significantly between the cohorts. Overall, the statistical analysis indicates that the two groups were comparable at baseline.

Among the six patients who survived to hospital discharge, the median age was 70.5 years (range: 55–81). This subgroup comprised four males and two females. Regarding airway management, five of these patients were managed with ETI, and one with an SGA. Their successful hospital length of stay ranged from 8 to 21 days.

Table 3 presents an evaluation of the effect of the airway management method on early survival within the study group. The model’s constant, which determines the odds of survival for the model’s reference values (the use of a supraglottic airway device in a 66-year-old female patient), was 1.03 and was statistically non-significant.

In the context of airway management methods, the variables (supraglottic airway device and endotracheal tube) demonstrate a difference in their impact on the outcome. The endotracheal tube, with a relative risk of 0.80 and a confidence interval of 0.33 to 1.88, does not demonstrate statistical significance (*p* = 0.598). This suggests that its effect on early survival is not statistically noticeable when compared to the supraglottic airway device.

Confounding variables, such as age and sex, were also analyzed. Age is statistically significant (*p* = 0.008) with an RR of 0.97 and a confidence interval of 0.95 to 0.99, indicating that with each advancing year of age, the probability of early survival decreases by an average of 3%. Sex (male), with a relative risk of 1.17 and a confidence interval of 0.54 to 2.50, is not statistically significant (*p* = 0.692), which means that differences in early survival between men and women are not statistically significant within this sample.

Based on the data analysis presented in Table 2, the hypothesis stating that the use of an endotracheal tube for airway management increases early survival in patients following out-of-hospital cardiac arrest is not supported by the available results.

Table 4 presents an evaluation of the impact of the airway management method on definitive survival within the study group. The relative risk for the endotracheal tube is 1.13, with a confidence interval of 0.37–4.53, and a *p*-value = 0.836. This indicates that there is no statistically significant difference in definitive survival between patients managed with an endotracheal tube and those who received a supraglottic airway device. An RR value greater than 1 suggests a slight increase in the likelihood of survival; however, the lack of statistical significance (a high *p*-value) implies that this result may be due to chance.

The model’s constant is 0.28, with a confidence interval of 0.08–0.79 and a *p*-value = 0.030, indicating statistical significance. This means that the baseline probability of definitive survival for 66-year-old females managed with a supraglottic airway device is relatively low (well below half of the patients).

Furthermore, the factors of sex and age did not significantly affect definitive survival.

In conclusion, the results do not confirm that the use of an endotracheal tube increases overall survival in patients following OHCA compared to the use of a supraglottic airway device.

**Figure 1:**
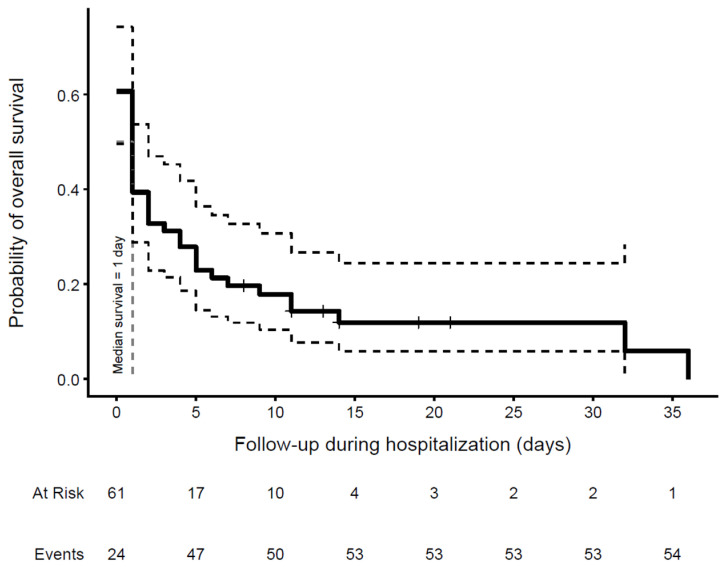
Kaplan–Meier curve for definitive survival during hospitalization in patients following out-of-hospital cardiac arrest.

Kaplan–Meier survival analysis revealed a marked reduction in the probability of definitive survival among patients admitted to the hospital following out-of-hospital cardiac arrest (OHCA), as shown in Figure 1. Beginning from day 1 of observation, the cohort included 37 individuals remaining at risk, with 37 events recorded, resulting in a survival probability of 0.39 (standard error [SE]: 0.06; 95% CI: 0.29–0.54). This trend persisted through day 7, when 13 patients remained at risk after 12 additional events occurred, leading to a further decline in survival probability to 0.20 (SE: 0.05; 95% CI: 0.12–0.33). By day 14, the number of individuals at risk decreased to six, with four events occurring, corresponding to a survival probability of 0.12 (SE: 0.04; 95% CI: 0.06–0.25). These observations indicate a high mortality rate during the initial period of hospitalization.

**Figure 2:**
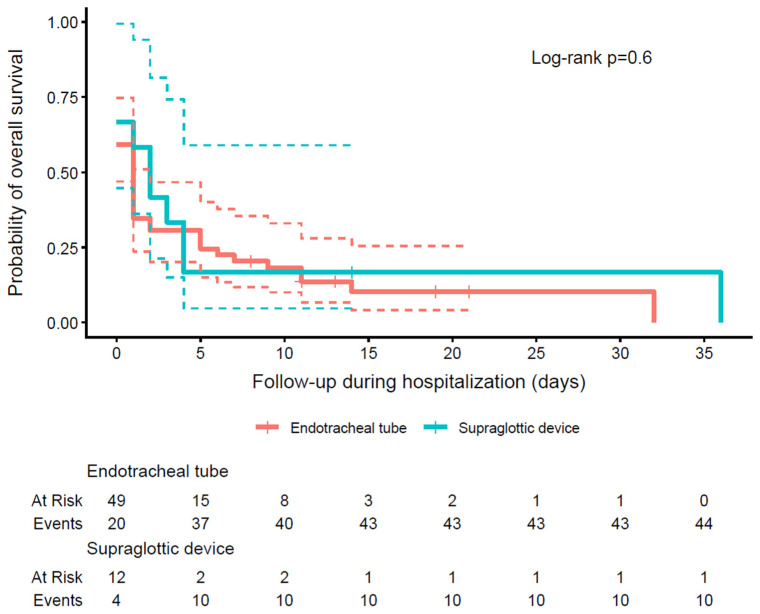
Kaplan–Meier curves for definitive survival during hospitalization, stratified by airway management device.

Following stratification by airway management method, as presented in Figure 2, distinct, albeit partially overlapping, survival patterns were observed. In the cohort of patients managed with an endotracheal tube, on day 1 of observation, 29 individuals remained at risk, with 32 recorded events, corresponding to a survival probability of 0.35 (SE: 0.07; 95% CI: 0.24–0.51). By day 7, the number of individuals at risk decreased to 11, with seven subsequent events, resulting in a decline in survival probability to 0.20 (SE: 0.06; 95% CI: 0.12–0.36). On day 14 of observation, four individuals were recorded at risk with four events, translating to a further decrease in survival probability to 0.10 (SE: 0.05; 95% CI: 0.04–0.26).

In the subgroup of patients managed with supraglottic airway devices, on day 1 of observation, eight individuals remained at risk, with five events, corresponding to a higher survival probability of 0.58 (SE: 0.14; 95% CI: 0.36–0.94). By day 7, this probability dropped sharply to 0.17 (SE: 0.11; 95% CI: 0.05–0.59), with two individuals remaining at risk and five events, after which it plateaued at this level until day 14, reflecting the absence of further events in this subgroup.

The log-rank test applied to compare the stratified survival curves yielded a *p*-value = 0.600, indicating a lack of statistically significant differences in overall survival between the endotracheal tube group and the supraglottic airway device group. This result suggests that within the analyzed dataset, the choice of airway management method did not have a significant impact on survival during hospitalization.

## 4. Discussion

The issue of how best to manage the airway in a patient with out-of-hospital cardiac arrest has generated significant debate within the emergency medicine community for years. For decades, endotracheal intubation was perceived as the inviolable “gold standard.” However, the latest data from large, multicenter trials are beginning to challenge this dogma. They suggest that in the chaos of prehospital interventions, it is the simplicity and speed of supraglottic airway devices that may genuinely improve patient prognosis.

An interesting starting point is the Taiwanese SAVE trial. Analyzing the outcomes of 936 patients, researchers noted that selecting intubation as the initial method did not increase the chances of sustained return of spontaneous circulation compared to supraglottic methods [5]. An important practical conclusion was drawn from this: in dense urban areas, where every second counts and response times are short, the invasiveness of intubation may simply not be justified.

Entirely different conclusions emerge from the secondary analysis of the Resuscitation Outcomes Consortium PRIMED trial data, encompassing an impressive cohort of 10,455 adults. In this cohort, 81.2% of patients received ETI, and 18.8% received SGA. The analysis demonstrated the superiority of intubation over supraglottic devices in terms of final outcomes [8]. Such a significant discrepancy may stem from differences in personnel experience—in cohort studies, intubation is often performed by the most experienced paramedics, which may bias the perception of the method itself.

A breakthrough in the discussion occurred with two massive randomized controlled trials published in 2018. The first, conducted by Henry E. Wang (the PART trial), included 3004 patients. It compared the laryngeal tube with ETI. The results indicated that a strategy utilizing LT was associated with significantly higher 72 h survival (18.3%) compared to the ETI strategy (15.4%) [4]. Concurrently, the AIRWAYS-2 trial was conducted in the UK, enrolling as many as 9289 patients. It compared the i-gel supraglottic airway with the endotracheal tube. This analysis showed no statistically significant difference in survival with a favorable neurological outcome at 30 days. However, this study highlighted a crucial problem: first-pass intubation success was significantly lower (70%) than with SGA (82%), which directly translates to time without chest compressions [9].

A new direction that may restore the appeal of intubation is the use of videolaryngoscopy. The latest meta-analyses indicate that the use of VL in prehospital settings significantly increases the first-pass success rate, eliminating the main criticism leveled against traditional intubation in the AIRWAYS-2 trial [10]. It is also worth recalling the French–Belgian CAAM trial, which, although focused on comparing ETI with bag–valve–mask ventilation, provided valuable safety data. It demonstrated that the intubated group experienced more frequent incidents of difficult airways and procedural failures [11]. Subsequent analyses indicate that an excessive focus on intubation can lead to dangerous interruptions in chest compressions, negating the benefits of better lung isolation.

Our own retrospective findings did not demonstrate a statistically significant difference in early or definitive survival between ETI and SGA. However, unlike the large, all-comer randomized trials such as PART and AIRWAYS-2, our findings must be interpreted within a highly specific clinical context. Several factors might explain the divergence or similarities between our results and the broader literature. First, our cohort demonstrated a strong local EMS preference for endotracheal intubation (49 ETI vs. 12 SGA). In our region, ETI is frequently performed by highly experienced paramedics or physician-led teams, which might mitigate the adverse effects of prolonged intubation attempts and chest compression interruptions seen in multicenter trials. Conversely, the selection of SGA might have been driven by specific emergency scenarios or utilized as a rescue device, introducing a degree of selection bias.

Furthermore, patient selection profoundly influences our results. By design, our study only included patients who successfully achieved ROSC and were admitted to the emergency department, excluding those whose resuscitation was unsuccessfully terminated on the scene. This creates substantial survivorship bias. Because airway interventions primarily influence gas exchange and hemodynamics during the active phase of chest compressions, restricting the analysis exclusively to ROSC patients potentially removes a critical part of the effect size we aimed to evaluate. Additionally, the lack of complete prehospital Utstein data (e.g., exact EMS response time, bystander CPR) introduces a high risk of residual confounding, as these unmeasured variables strongly dictate the likelihood of achieving ROSC independent of the airway device used.

Therefore, while our study aligns with the recent trends suggesting no clear superiority of ETI over SGA, our small sample size and the resulting lack of statistical power preclude any definitive statements regarding clinical equivalence. Although intubation remains the method offering the best protection against gastric content aspiration, the choice of prehospital airway management should remain individualized. This decision must be driven by operator experience, local EMS protocols, and the critical need to maintain high-quality chest compressions, until more robust, adequately powered prospective data become available.

### Limitations of the Study

The presented results should be interpreted in light of several significant limitations. First and foremost, the study is based on a retrospective analysis of medical records from a single emergency department. Such a design imposes a strict reliance on the quality of prehospital and hospital medical documentation; we had no control over what was recorded during the high-stress environment of resuscitation efforts. Furthermore, by analyzing only patients who achieved ROSC and were transported to the hospital, we excluded cases where resuscitation was unsuccessfully terminated at the scene, which inherently introduces selection bias and narrows our perspective.

Another limitation is the size of the study group. Following the application of inclusion and exclusion criteria, the final cohort was reduced to *n* = 61, which results in low statistical power, particularly regarding survival to hospital discharge. Because only six patients survived to discharge, attempting to build advanced regression models for this parameter carries a high risk of statistical error. Therefore, our conclusions regarding definitive survival should be considered preliminary trends and signals for further research rather than robust evidence.

Finally, it is crucial to address the lack of certain core data points required by the Utstein style. Specifically, the available prehospital documentation did not reliably allow for the precise determination of the Emergency Medical Services response time, the initiation of bystander CPR, the initial cardiac rhythm, or the use of an AED. As these factors—particularly EMS response time and bystander CPR—are well-established, independent predictors of OHCA survival, the inability to include them in our statistical analysis represents a notable limitation. Incorporating only fragmentary data would severely compromise the validity of the results. Consequently, the observed impact of the airway management method (ETI vs. SGA) could not be statistically adjusted for these critical prehospital timelines, which may confound the outcomes.

Because this is a single-center study, the specific nature of operations within our local EMS system means our findings cannot be uncritically extrapolated to the general population or other regions. Future prospective, multicenter studies with integrated dispatch and comprehensive prehospital registries are necessary to fully evaluate these interactions and overcome these limitations.

## 5. Conclusions

In this small, retrospective cohort of patients who achieved ROSC, no statistically significant difference in in-hospital survival was observed between endotracheal intubation and the use of supraglottic airway devices. However, because the study is underpowered and carries a high risk of residual confounding due to missing prehospital variables, this absence of statistical significance should not be interpreted as definitive evidence of clinical equivalence. Consequently, we cannot definitively conclude that the prehospital airway management method lacks an independent impact on survival. Until further adequately powered, prospective data are available, the decision regarding the method of airway management should remain individualized. This choice must primarily consider the operator’s experience and competence, the availability of equipment, and the critical need to minimize interruptions in chest compressions while executing key elements of advanced life support.

## Figures and Tables

**Table 1:** Characteristics of the study population.

*Characteristics*	*N*	*Mdn*	*Q1, Q3*	*Min–Max*	*N%*
Age, years	61	66.00	54.00, 75.00	19.0–90.0	100%
Female	16	n/a	n/a	n/a	26.23%
Male	45	n/a	n/a	n/a	73.77%
Death/mortality	55	n/a	n/a	n/a	90.16%
Early survival	31	n/a	n/a	n/a	50.82%
Definitive survival	6	n/a	n/a	n/a	9.84%
Endotracheal tube (ETT)	49	n/a	n/a	n/a	80.33%
Supraglottic airway device (SGA)	12	n/a	n/a	n/a	19.67%

N—number of cases in the group; Mdn—median; Q1—first quartile (25th percentile); Q3—third quartile (75th percentile); Min—minimum value; Max—maximum value; n/a—not applicable.

**Table 2:** Baseline characteristics and clinical outcomes of the study population categorized by prehospital airway management strategy.

Characteristics	Total Cohort (*n* = 61)	ETI Group (*n* = 49)	SGA Group (*n* = 12)	*p*
Age (years), Mdn (Q1–Q3)	66.0 (54.0–75.0)	66.0 (52.0–76.0)	65.5 (56.8–71.5)	0.710
Male sex, *n* (%)	45 (73.8%)	38 (77.6%)	7 (58.3%)	0.270
Early survival, *n* (%)	31 (50.8%)	24 (49.0%)	7 (58.3%)	0.749
Definitive survival, *n* (%)	6 (9.8%)	5 (10.2%)	1 (8.3%)	>0.99

*n*—number of cases in the group; Mdn—median; Q1—first quartile (25th percentile); Q3—third quartile (75th percentile).

**Table 3:** Evaluation of the impact of the airway management method on early survival within the study group.

Explanatory Variables	Early Survival		
	RR	95% CI	*p*
(Constant)	1.03	0.42–2.48	0.949
Exposure			
Supraglottic airway device	Reference category		
Endotracheal tube	0.80	0.33–1.88	0.598
Confounding variables			
Age	0.97	0.95–0.99	0.008
Sex [male]	1.17	0.54–2.50	0.692

RR—relative risk, 95% CI—95% confidence interval, *p*—*p*-value of the statistical test.

**Table 4:** Evaluation of the impact of the airway management method on definitive survival within the study group.

Explanatory Variables	Definitive Survival		
	RR	95% CI	*p*
(Constant)	0.28	0.08–0.79	0.030
Exposure			
Supraglottic airway device	Reference category		
Endotracheal tube	1.13	0.37–4.53	0.836
Confounding variables			
Age	1.02	0.99–1.06	0.270
Sex [male]	0.85	0.32–2.42	0.741

RR—relative risk, 95% CI—95% confidence interval, *p*—*p*-value of the statistical test.

## Data Availability

The datasets used and/or analyzed during the current study are available from the corresponding author on reasonable request.

## Abbreviations

The following abbreviations are used in this manuscript:

ALS	Advanced Life Support
CI	Confidence Interval
CPR	Cardiopulmonary Resuscitation
ED	Emergency Department
EMSs	Emergency Medical Services
ERC	European Resuscitation Council
ETI	Endotracheal Intubation
ETT	Endotracheal Tube
ICD-10	International Classification of Diseases, Tenth Revision
LMA	Laryngeal Mask Airway
LT	Laryngeal Tube
Max	Maximum Value
Mdn	Median
Min	Minimum Value
N	Number of Cases in the Group
n/a	Not Applicable
OHCA	Out-of-Hospital Cardiac Arrest
*p*	*p*-value of the Statistical Test
Q1	First Quartile (25th percentile)
Q3	Third Quartile (75th percentile)
ROSC	Return of Spontaneous Circulation
RR	Relative Risk
SD	Standard Deviation
SE	Standard Error
SGA	Supraglottic Airway Device
VL	Videolaryngoscopy
